# Idiosyncratic drug-induced liver injury related to use of novel selective androgen receptor modulator RAD140 (Testalone): a case report

**DOI:** 10.1186/s13256-023-03847-8

**Published:** 2023-03-29

**Authors:** Michael Ladna, Kellee Taylor, Adnan Bhat, Bahram Dideban

**Affiliations:** 1 Department of Hospital Medicine, University of Florida, Gainesville, FL USA; 2 Department of Medicine, University of Florida, 1600 SW Archer Road, Room 4102, Gainesville, FL 32610 USA

**Keywords:** Drug-induced liver injury, Acute liver disease, Drug toxicity

## Abstract

**Background:**

RAD140 (Testalone) is a novel selective androgen receptor modulator with very limited data currently available on adverse effects related to this compound. The first-in-human phase 1 trial was recently published and did report a significant proportion of elevated aspartate aminotransferase, alanine transaminase, and total bilirubin among the test subjects. RAD140 may be associated with an idiosyncratic drug-induced liver injury. It is easily purchased online as a workout supplement. Given its ease of use from being an oral formulation, and not requiring a physician’s prescription, its use among the young male population will likely rise. Clinicians should ask about the use of RAD140, and other workout supplements, in young men presenting with acute liver injury.

**Case presentation:**

We present the case of a 26-year-old Caucasian male without any significant past medical history who presented with nausea, vomiting, severe right upper quadrant abdominal pain, and jaundice from acute liver injury. Extensive inpatient workup did not reveal a definite cause for his liver injury other than the use of a novel selective androgen receptor modulator called RAD140 (Testalone). He was treated with supportive care and discharged after short hospitalization. He was instructed to stop RAD140, which he reported compliance with, and on 2-month follow-up his liver function panel had normalized without recurrence of any symptoms.

**Conclusion:**

Novel selective androgen receptor modulators such as RAD140 may be associated with idiosyncratic drug-induced liver injury. Workup of new liver injury in young and middle-aged males should involve asking about use of these novel compounds, for if missed and use continues, it can likely lead to fulminant liver failure or decompensated liver cirrhosis.

## Introduction

Young men are using oral androgen agonists more often, and there is a constant influx of these unregulated agents, which can be easily purchased over the internet. The adverse effects of these substances are not well researched. Unregulated, these substances may contain active compounds and contaminants ultimately associated with adverse effects, including hepatotoxicity. The use of these agents will likely increase given their ease of use compared with traditional testosterone therapy, which is intramuscular and available by prescription only. It is important for the clinician to be aware of these substances and the potential adverse effects, such as acute liver injury, to ensure the appropriate diagnosis can be made and the patient can be instructed to stop the use of these substances to prevent further liver injury, and ultimately fulminant liver failure. This case also highlights the value of thorough history taking, which can be difficult to perform in the modern age of productivity-based medical practice.

## Case presentation

A 26-year-old Caucasian male without any past medical history presented to emergency department with chief complaint of yellow skin and eyes, nausea, nonbloody vomiting, and severe right upper quadrant abdominal pain. These symptoms first started 9 days prior and had been getting progressively worse. The abdominal pain was intermittent and associated with dark, tea-colored urine and constipation. He denied use of any acetaminophen recently, alcohol use, illicit drug use, or personal/family history of hereditary hemochromatosis, Wilson’s disease, or celiac disease. He did report use of a new workout supplement called RAD140 (Testalone). He had previously been using a powdered version of the compound but switched to a liquid version approximately 2 months ago. Physical exam was significant for jaundice with scleral icterus and epigastric, as well as right upper quadrant abdominal, tenderness to palpation without rigidity, rebound, or guarding.

Laboratory work revealed transaminitis with aspartate transaminase (AST) of 51 IU/L, alanine transaminase (ALT) of 243 IU/L, and hyperbilirubinemia with total bilirubin of 4.9 mg/dL. Lactate dehydrogenase (LDH), alkaline phosphatase, and gamma-glutamyltransferase (GGT) were all within norms. Computed tomography (CT) of the abdomen and pelvis showed normal appearing liver and gallbladder. Right upper quadrant ultrasound was unremarkable, revealing a liver with homogeneous echotexture without focal parenchymal lesion, a common bile duct of 8 mm without biliary ductal dilatation, and a normal gallbladder without evidence of cholecystitis. Antismooth muscle, antimitochondrial, liver–kidney microsomal, antitissue transglutaminase, anti-endomysial, and gliadin immunoglobulin (Ig)G antibodies and antinuclear antibodies (ANA) were all negative. IgG, IgA, IgM, and ceruloplasmin all within normal limits. Tested negative for hepatitis A, B, and C. Acetaminophen level was undetectable. Hemochromatosis gene testing was negative. Research into RAD140 revealed that it was a novel selective androgen receptor modulator (SARM) called Testolone and has been associated with drug-induced liver injury (DILI) in case reports in the available literature. Due to extensive negative workup and discussion with hepatology colleagues, it was determined that the most likely cause of DILI was either RAD140 itself or another unknown but active compound or contaminant in the over-the-counter product. Treatment was supportive care via pain control, nausea control, and hydration. He was counseled to stop taking RAD140, which he was agreeable to.

He was hospitalized for a total of 4 days and discharged home. His symptoms of abdominal pain, nausea, and vomiting resolved during the hospitalization. Although the transaminitis improved while inpatient, the hyperbilirubinemia trended up. Per hepatology, this was attributed to being part of the typical course of DILI and he was safe to be discharged. Repeat outpatient laboratory work revealed normalization of AST, ALT, and total bilirubin 1 month after discharge. He followed up with hepatology in the clinic 2 months after discharge and reported cessation of RAD140 medication and was asymptomatic at that time without recurrence of abdominal pain, nausea, vomiting, or jaundice.

## Discussion and conclusions

RAD140 was first discovered by Radius Health, Inc in 2010. It belongs to a group called selective androgen receptor modulators (SARMs), which are nonsteroidal, orally active molecules developed to bind androgen receptors strongly in targeted tissues (muscle and bone) and weakly in genital tissues, with the goal of treating sarcopenia and osteoporosis while minimizing worsening of prostate disease in men and virilization in women [1]. SARMs are widely available on the internet as nutritional supplements, despite not being approved for human consumption. One study of various SARM products found that only 23/44 (53%) of the products tested contained 1 or more SARM, 17 (39%) contained another unapproved drug, and 4 (9%) did not contain any active compound [2]. Only one case report from early 2020 documented DILI with RAD140/Testolone [3]. There is also a single case report documenting acute myocarditis associated with RAD140 [4].

The first in-human phase 1 clinical trial of RAD140 was published in January 2022 and included a total of 22 postmenopausal women with stage IV, ER^+^/HER2^−^ breast cancer. The most common adverse effects were elevated AST (59.1%), ALT (45.5%), total bilirubin (27.3%), and hypophosphatemia (22.7%), as well as dehydration, vomiting, anorexia, and weight loss (27.3% each). The acceptable safety profile was at doses of 50–100 mg daily, with the 150 mg daily dose group having significantly more hyperbilirubinemia and hypophosphatemia. All these adverse events were reversible, and there were no treatment-related deaths or serious adverse events (AE) leading to study withdrawal [5].

Owing to frequent contamination and mislabeling of nutritional supplements, it is difficult to conclude whether this case of DILI was due to the active compound RAD140, or another active component or contaminant. The wide availability of these compounds to retail consumers should encourage physicians who encounter acute liver injury of undetermined etiology to inquire about the recreational use of these novel compounds. Individuals are at high risk of taking excessive doses or dosing too frequently since data on safe doses has only just recently been published [5]. The use of a liquid form of RAD140 via dropper, as was the case with our patient, likely made administration of an accurate dose more challenging, leading to unintentional toxic dosing of the compound.

Patients with acute liver injury require a thorough diagnostic workup to reveal the pathology. Etiologies such as autoimmune hepatitis, Wilson’s disease, alcoholic hepatitis, Tylenol toxicity, acute and chronic viral hepatitis, primary sclerosing cholangitis, and primary biliary cholangitis must be ruled out prior to making a diagnosis of idiosyncratic DILI. DILI not associated with Tylenol use is typically managed with supportive care, immediate cessation of the insulting compound, and referral to liver transplantation if the patient presents with fulminant acute liver failure or there is worsening of liver injury, despite cessation of the insulting compound. Our patient was managed with supportive care and had resolution of DILI after cessation of RAD140 use.

## Data Availability

Not applicable.
